# Quantifying and contextualizing the impact of bioRxiv preprints through automated social media audience segmentation

**DOI:** 10.1371/journal.pbio.3000860

**Published:** 2020-09-22

**Authors:** Jedidiah Carlson, Kelley Harris

**Affiliations:** 1 Department of Genome Sciences, University of Washington, Seattle, Washington, United States of America; 2 Computational Biology Division, Fred Hutchinson Cancer Research Center, Seattle, Washington, United States of America; University of Minnesota, UNITED STATES

## Abstract

Engagement with scientific manuscripts is frequently facilitated by Twitter and other social media platforms. As such, the demographics of a paper's social media audience provide a wealth of information about how scholarly research is transmitted, consumed, and interpreted by online communities. By paying attention to public perceptions of their publications, scientists can learn whether their research is stimulating positive scholarly and public thought. They can also become aware of potentially negative patterns of interest from groups that misinterpret their work in harmful ways, either willfully or unintentionally, and devise strategies for altering their messaging to mitigate these impacts. In this study, we collected 331,696 Twitter posts referencing 1,800 highly tweeted bioRxiv preprints and leveraged topic modeling to infer the characteristics of various communities engaging with each preprint on Twitter. We agnostically learned the characteristics of these audience sectors from keywords each user’s followers provide in their Twitter biographies. We estimate that 96% of the preprints analyzed are dominated by academic audiences on Twitter, suggesting that social media attention does not always correspond to greater public exposure. We further demonstrate how our audience segmentation method can quantify the level of interest from nonspecialist audience sectors such as mental health advocates, dog lovers, video game developers, vegans, bitcoin investors, conspiracy theorists, journalists, religious groups, and political constituencies. Surprisingly, we also found that 10% of the preprints analyzed have sizable (>5%) audience sectors that are associated with right-wing white nationalist communities. Although none of these preprints appear to intentionally espouse any right-wing extremist messages, cases exist in which extremist appropriation comprises more than 50% of the tweets referencing a given preprint. These results present unique opportunities for improving and contextualizing the public discourse surrounding scientific research.

## Abstract 2

Analysis of Twitter data for 1,800 bioRxiv preprints to classify the online communities engaging with biological research enables the tracing of each preprint's exposure to various academic disciplines and non-specialist audiences, potentially indicating downstream scientific and societal impacts, and revealing that some preprints receive extraordinarily high engagement from white nationalist communities.

## Introduction

In the last decade, scientists have flocked to Twitter and other online social media platforms to share their research, connect with colleagues, and engage with the public [1]. The enthusiastic adoption of social media by some researchers has dramatically altered how scientific publications are diffused throughout the scientific community and bridged into the public forum, leading some in the research community to consider social media as a vital tool for public engagement and scientific communication [2]. Metrics gleaned from social media data (commonly known as “altmetrics”) can provide a nearly instantaneous readout of a paper’s exposure across broad cross-sections of experts and lay audiences alike [3]. Though some have speculated that “social media buzz” about research has the potential to be highly superficial and not necessarily indicative of lasting societal or scientific impact [4–6], others argue that social media attention might provide an immediate indicator of an article’s potential downstream impacts, including (1) predicting—and perhaps even causally increasing—the number of times one’s research is cited in the scientific literature [7,8]; (2) facilitating engagement with news media and public audiences [9,10]; (3) fostering networking, collaboration, and career development opportunities [2]; and (4) improving the likelihood of receiving grant funding [11].

The most popular measure of social media attention currently in use is the Altmetric Attention Score, which is calculated as a weighted average of nearly every instance in which an article is referenced on social media sites like Twitter, Facebook, and Reddit, along with mentions in news articles, Wikipedia pages, and policy documents. Metrics like the Altmetric Attention Score, however, have been criticized for failing to provide the appropriate context necessary for evaluating societal impact [12,13]. Altmetric readily acknowledges that their Attention Score alone is insufficient for evaluating a paper’s scope of impact: In their introduction to the list of papers that received the top 100 Attention Scores of 2019, Altmetric cautions that “the only theme that many of these papers have in common is their ability to start conversations…the ranking has no bearing on the quality or impact of the research itself” [14]. Identifying these “conversation starter” papers is certainly a valuable indicator of research that might lead to tangible impacts on the broader public, but if altmetrics are to be harnessed to meaningfully measure societal impact, we must move beyond the simple question of *how much* attention an article receives on social media, and prioritize questions of context: *Who* is engaging with the article, *what* they are saying about it, and *when* and *where* they are doing so [13,15]. In particular, identifying characteristics of nonacademic social media audiences is an important first step in understanding potential societal impacts [16].

Altmetric currently provides cursory classifications of Twitter users who have engaged with an article, sorting the overall audience into 4 basic categories: 3 describe different groups of academic/scientific stakeholders (“scientists,” “practitioners,” “science communicators”), and a fourth, “members of the public,” encompasses all others who do not fit into one of the other categories [17]. In a blog post written for Altmetric [16], Haustein, Toupin, and Alperin explain that Altmetric’s classification algorithm primarily relies on how individual users self-identify in their Twitter bios. Because Twitter bios are limited to 160 characters, Altmetric’s classification scheme is highly sensitive to the inherent sparsity and noise of this information. Ultimately, Altmetric acknowledges that users whose bios lack keywords that classify them as academic/scientific are relegated to the “members of the public” category, even if those users are, in fact, part of the scientific research community [16].

Fortunately, contrary to Altmetric’s claims that individual Twitter bios are “pretty much the only” [16] source of information for classifying users, we are not restricted to learning about audience characteristics from individual users’ self-descriptions (or lack thereof). A cornerstone of sociological research is the principle of network homophily, which states that individuals tend to connect and engage within a social network of others who share similar characteristics [18]. Online social media is no exception: Patterns of network homophily have been demonstrated in numerous studies of Twitter users [19–23], and there is evidence to suggest that the homophily of an individual’s online connections generally mirrors their face-to-face networks [24]. Recent studies have applied this principle to the study of altmetrics and demonstrated that deeper contextualization of a publication’s audience on social media can be achieved by examining various aspects of how individual users are networked with others on Twitter [13,25]. More specifically, network homophily enables the identification of various characteristics of an individual Twitter user based on the self-descriptions of the accounts connected with that individual on Twitter [20,26].

In this study, we present a framework for segmenting a scholarly article’s audience on Twitter (specifically, the set of users that tweeted or retweeted a link to the article) into granular, informative categories inferred through probabilistic topic modeling of metadata collected from each user’s network of followers. Probabilistic topic modeling is a powerful and flexible method for revealing the granular composition of all sorts of complex datasets—not only the present application of text mining [27], but also inference of population ancestry using genetic markers [28], computer vision [29], and many other areas of study. With this approach, we analyze the Twitter audiences for a selection of 1,800 highly tweeted preprints (encompassing over 330,000 tweets in total) across a variety of biological research disciplines. We show that each article’s audience on social media is characterized by a unique composition of online communities, both within academia and among diverse nonspecialist audiences. The audience classifications inferred with our topic modeling framework thus provide valuable context for interpreting altmetrics and providing initial traces of potential societal impacts.

We highlight 3 ways in which these inferred audience demographics can serve to enhance interpretation of altmetrics. First, we show that this approach produces more accurate quantification and classification of the various academic audience sectors that express interest in a particular research output. Second, we identify rich, qualitative characteristics of nonacademic audiences that inform potential societal impacts. Third, we explore how politically engaged audiences are interacting with research. Because detailed context is such an important aspect of interpreting altmetrics, we have compiled an online portal showcasing summary data across all preprints analyzed along with interactive visualizations detailing the various dimensions of audience characteristics for individual preprints, available at http://carjed.github.io/audiences.

Our analyses also reveal a subset of preprints that attract a great deal of attention from audience sectors associated with far-right ideologies, including white nationalism. These communities appear to be especially active in their engagement with preprints concerning the genetic architecture of behavioral traits, human population genetics, and ancient DNA research, and the neurological and physiological variation across sexes and genders, among a plethora of other topics. In some cases, these extremist-affiliated users comprise over half of the total Twitter audience engaging with an article, providing concrete evidence to support concerns about racist and sexist misappropriation of research that have been expressed by academic organizations [30], news media [31], and scientists themselves [32]. We discuss how stakeholders in the scientific community can use these audience demographics to understand the social and political implications of their work and assert their expertise to guide public science literacy in the era of social media.

## Results

### Data collection

We used Rxivist, a service that indexes article-level metrics for preprints posted to the bioRxiv preprint repository [33], to identify 1,800 preprints posted between November 19, 2013, and February 10, 2020, that received more than 50 tweets and were among the most highly downloaded or tweeted according to Rxivist’s rankings (Materials and methods). The number of tweets per preprint ranged as high as 3,794 tweets, with a median of 113 tweets. We focused on manuscripts in the bioRxiv preprint repository rather than peer-reviewed journals for the following reasons: (1) bioRxiv preprints are organized into 27 different categories, covering a broad cross-section of topics in the biological sciences, whereas most peer-reviewed journals are specific to a single area of research and/or do not discretely categorize papers; (2) metadata for bioRxiv preprints is readily retrievable through the open-source Rxivist platform, enabling a relatively unbiased selection of highly tweeted research from the same source; (3) bioRxiv preprints are fully open-access and freely available to read by anyone, unlike many peer-reviewed journals that are wholly or partially restricted by paywalls; and (4) unlike many peer-reviewed journals, bioRxiv does not highlight or promote certain preprints over others, nor are preprints typically promoted to the news media via press releases, creating a more even playing field for preprints to organically garner attention on social media.

### Topic modeling for audience sector classification

Given a preprint and the list of Twitter users that tweeted or retweeted a link to it, we are primarily interested in identifying latent characteristics of this Twitter audience (e.g., occupation, personal interests, hobbies, political affiliation, etc.) and quantifying the relative abundance of these various audience sectors. Under the principle of network homophily, we assume that individual-level characteristics of a user can be accurately inferred from information aggregated across that user’s network of followers, namely, the self-descriptions provided by the user’s followers in their Twitter biographies. Therefore, for each preprint, we collected information about each user that tweeted or retweeted a link to that preprint, then queried the Twitter API (application programming interface) to collect metadata for each of these user’s followers (Materials and methods). For each user, we then compiled their followers’ biographies and screen names into a single vector of words (including emoji and hashtags), which we refer to as a “follower document.” Our subsequent analyses of these follower documents used a “bag of words” approach, that is, the order in which words occurred in each document (and the specific follower they originated from) is ignored. We refer to the collection of follower documents for a given preprint as the “audience corpus.”

For each preprint, we extracted information about the underlying audience sectors—or “topics”—by applying a latent Dirichlet allocation (LDA) model [27] to the audience corpus of associated follower documents (Materials and methods). The LDA model assumes that each audience corpus consists of *K* distinct underlying audience sectors, such that each follower document can be represented probabilistically as a mixture of these *K* audience sectors, with each audience sector represented by a collection of words that frequently co-occur within the same latent topic. We expect that each of these representations conveys semantic meaning about the characteristics of a given audience sector, e.g., a topic represented by the keywords “professor,” “university,” “genetics,” “population,” and “evolution” likely reflects an audience sector of population/evolutionary geneticists.

### Inferring the diversity of academic audiences on social media

**Fig 1:**
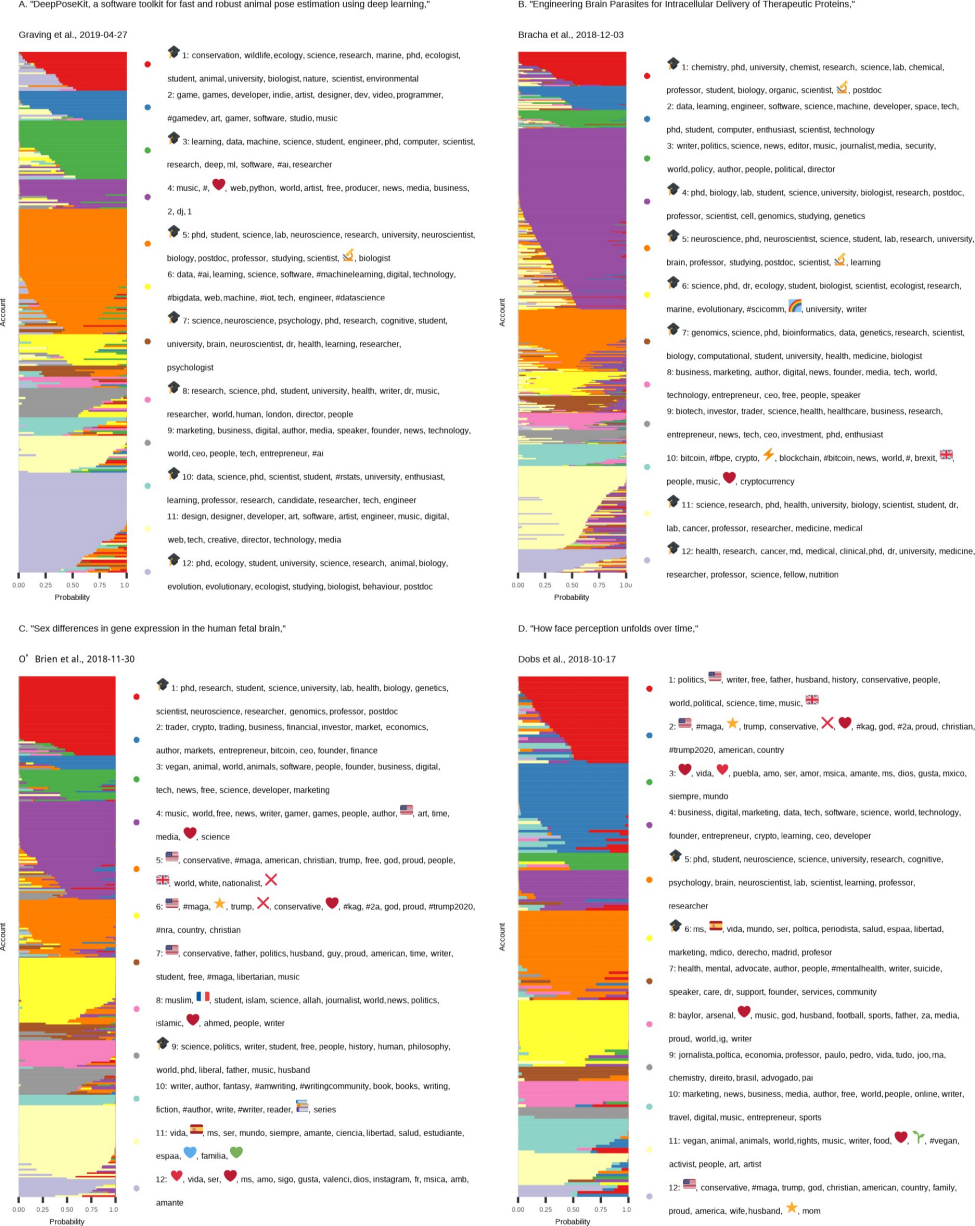
Topic modeling output for 4 selected preprints. Each panel shows the LDA topic modeling results for one of 4 selected preprints: **(A)** “DeepPoseKit, a software toolkit for fast and robust animal pose estimation using deep learning,” by Graving and colleagues. **(B)** “Engineering Brain Parasites for Intracellular Delivery of Therapeutic Proteins,” by Graving and colleagues. **(C)** “Sex differences in gene expression in the human fetal brain,” by O’Brien and colleagues. **(D)** “How face perception unfolds over time,” by Dobs and colleagues. In each panel, every account that tweeted about that paper is represented by a horizontal stack of 12 colored segments that represent the account’s estimated membership probability in each of the 12 inferred audience sectors. The top 15 keywords, hashtags, or emoji associated with each topic are shown in the corresponding legend to the right of each panel. Topics inferred to correspond to academic audience sectors are indicated with a 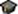 emoji at the beginning of the list of associated keywords. Data for the information depicted in this figure are available at https://github.com/carjed/audiences, and interactive versions of this figure for each of the 1,800 preprints analyzed can be accessed at https://carjed.github.io/audiences. LDA, latent Dirichlet allocation.

Examples of the LDA topic modeling results for 4 selected preprints are shown in **Fig 1**, in which each user that referenced a given preprint is displayed as a stack of horizontal bars segmented into *K* colors representing the estimated membership fraction in each audience sector. This visualization strategy is analogous to the de facto standard of visualizing inferred genetic ancestry of individuals as implemented in the popular *STRUCTURE* program, which uses the same underlying statistical model as LDA [34,35]. We selected these particular preprints because they represent a variety of scientific disciplines covered on bioRxiv (animal behavior, synthetic biology, genomics, and neuroscience), and their audience sectors exhibit a wide range of academic and nonacademic communities. For each preprint, we classified each of the inferred audience sectors as “academic” or “nonacademic,” flagging topics whose top keywords include a combination of words indicating an academic affiliation (such as “PhD,” “university” or “professor”) as “academic audience sectors” and classifying all other topics as “nonacademic audience sectors.”

As shown in **S1 Fig**, many preprints had academic audience sectors that generally aligned with the bioRxiv category under which the preprint was classified. Overall, we found 96% of preprints had at least 1 audience sector concordant with the focal bioRxiv category. The median fraction of the audience that was associated with these concordant sectors was 70%. These results demonstrate that our method captures relevant qualitative information about each preprint’s Twitter audience. For example, the preprint “DeepPoseKit, a software toolkit for fast and robust animal pose estimation using deep learning,” by Graving and colleagues, was submitted to bioRxiv in the “animal behavior and cognition” category [36], and topics 1 and 12 included the keyword “animal,” indicating a match for this focal category (also note that topics 1 and 12 for this preprint included the keyword “ecology”) (**Fig 1A**). “Engineering Brain Parasites for Intracellular Delivery of Therapeutic Proteins,” by Bracha and colleagues, was submitted to bioRxiv in the “synthetic biology” category [37]—though we did not identify any topics containing the keyword “synthetic,” several topics indicated a match with academic disciplines that appear related to this subject, including neuroscience (topic 5) and medical research (topics 11 and 12) (**Fig 1B**). “Sex differences in gene expression in the human fetal brain,” by O’Brien and colleagues, was submitted to bioRxiv in the “genomics” category [38], with topic 1 indicating a match with the focal category (**Fig 1C**). “How face perception unfolds over time,” by Dobs and colleagues, was submitted to bioRxiv in the “neuroscience” category [39], with topic 5 indicating a match with the focal category (**Fig 1D**).

**Fig 2:**
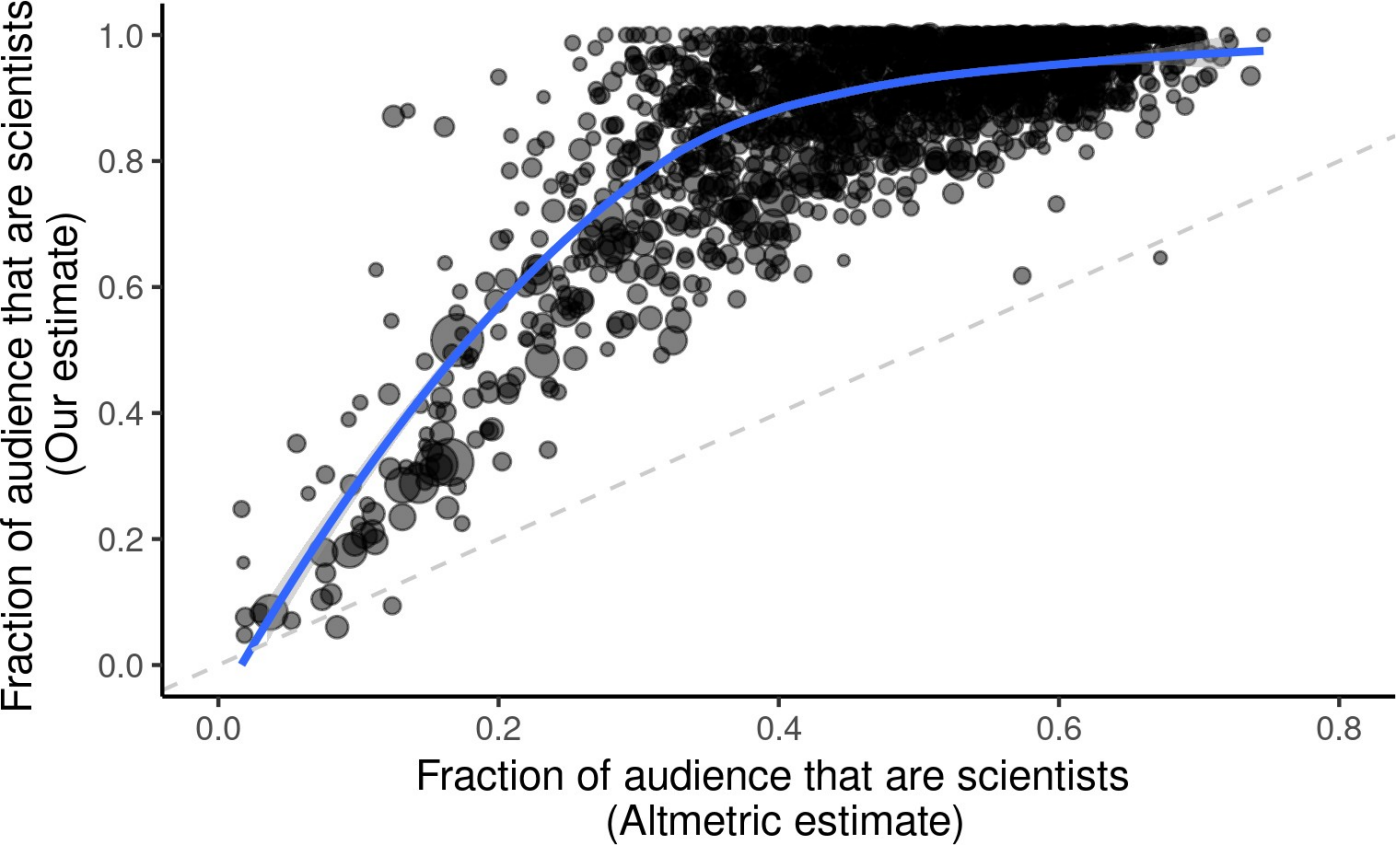
Comparison of academic audience fractions estimated by Altmetric versus our estimates. Each point represents an individual preprint, with Altmetric’s estimated academic audience fraction shown on the x-axis and the academic audience fractions estimated through our topic modeling approach shown on the y-axis. The loess-smoothed curve fit to the data indicates a nonlinear relationship between these 2 sets of estimates. The size of each point indicates the total number of tweets referencing that preprint. Data for the information depicted in this figure are available at https://github.com/carjed/audiences, and an interactive version of this figure can be accessed at https://carjed.github.io/audiences.

One way we can attempt to coarsely quantify the broader societal impact of a preprint using these data is to compare the relative engagement from academic versus nonacademic audiences on social media [40], similar to how Altmetric provides an estimated demographic breakdown of users tweeting about a paper [17]. For each preprint, we estimated the fraction of the audience falling into any of the inferred academic audience sectors and nonacademic audience sectors (Materials and methods). We found that the academic audience sectors typically comprised the majority of a preprint’s audience—across the 1,800 preprints analyzed, 95.9% had a majority audience consisting of academic audience sectors, as inferred by our method (**Fig 2, S2 Fig**). We also observed a slight negative correlation (Spearman’s ρ = −0.233) between our academic audience fraction estimates and the total number of tweets, suggesting that a preprint’s attention on Twitter is somewhat influenced by its exposure and penetration into nonacademic audiences, but this relationship is nonlinear and may be driven by a handful of outliers that received many more tweets than the typical preprint in our dataset (**S3 Fig**).

Our estimates of academic audience fractions were consistently higher than the fraction of users inferred to be “scientists,” “science communicators,” or “practitioners” by Altmetric, with our method estimating a higher proportion of academic-affiliated users for all but 3 of the 1,800 preprints (**Fig 2**). Several lines of evidence suggest that the primary reason for this discrepancy is that Altmetric systematically fails to properly classify many scientists, rather than our method systematically overestimating the sizes of the academic audiences. First, we refer to Altmetric’s description of their classification scheme, which states “the ‘members of the public’ category by Altmetric absorbs all users without keywords that classify them as scientists, science communicators or practitioners” [16]. In our data, we observe approximately 8% of Twitter users have completely empty bios, and many more likely have sparse or uninformative bios that Altmetric cannot unambiguously classify. Coupled with our finding that Altmetric’s academic audience estimates have an upper bound of approximately 0.75 for the preprints we analyzed (**Fig 2**), this suggests that each preprint has a substantial baseline fraction of users that Altmetric will always classify by default as “members of the public” because of the lack of information in their individual bios. Second, if Altmetric is indeed biased towards classifying users as “members of the public,” we would expect that their estimates converge with ours for preprints with the smallest academic audiences. Indeed, we found that the 2 estimates of academic audience fractions were generally well correlated (Spearman’s ρ = 0.5672), but this relationship was nonlinear, with the strongest concordance observed for preprints found to have relatively small academic audience fractions by both methods (**Fig 2**). Third, we found that Altmetric’s estimates of academic audiences comprised a majority for only 13 (27.1%) of the 48 preprints analyzed in the “scientific communication and education” category (**S2 Fig**). Nearly all preprints we analyzed in this category concern topics that we expect have little relevance outside of academia, including several surveys of academic personnel, investigations of biases in publication/peer review processes, assessment of grant-funding practices, and scientometrics and altmetrics. In contrast, our method consistently estimated that academic sectors comprised a majority of the audiences for all these preprints, with a median academic audience fraction of 92.6% and a minimum of 61%.

We acknowledge that the ideal strategy for comparing our method against Altmetric’s would be to manually classify each of the 331,696 users in our dataset and test the 2 methods’ accuracy against this ground truth. Though performing this manual validation is not feasible at scale, we did so for one preprint, “Shake-it-off: A simple ultrasonic cryo-EM specimen preparation device,” [41] as a proof-of-concept to demonstrate that our method is likely a more accurate classifier of academic-affiliated accounts than Altmetric’s method. Our rationale for selecting this preprint for validation was that it received relatively few tweets that needed to be manually annotated but had high discordance between our estimated academic audience fraction (100%) and that estimated by Altmetric (39.8%). In addition, the title of this preprint contains a pithy reference to the title of the song “Shake it Off” by pop singer Taylor Swift—this was not a coincidence, as the most retweeted tweet about the preprint was posted by the first author, John Rubenstein, who described their newly devised cryogenic electron microscopy device using a parody of a line from the chorus of Swift’s song:

Sprayer's gonna spray spray spray…

*Shake-it-off: A simple ultrasonic cryo-EM specimen preparation device*.

(https://twitter.com/RubinsteinJohn/status/1126553381374439424)

This is relevant because papers with "amusing, quirky or off-beat" research or those that contain buzzwords in the title are often presumed to attract greater attention from the public [4], so we reasoned that this preprint in particular would align with the null hypothesis that Altmetric's estimate of a majority nonacademic audience was correct for this preprint.

At the time we collected data for this preprint, Altmetric had indexed 88 unique users that had tweeted about this paper and estimated that 35 of these users were either scientists (33), practitioners (1), or science communicators (1), and the remaining 53 were members of the public. Our method only analyzed 73 of these accounts (15 were private accounts or had fewer than 5 followers and were excluded), but among these, all 73 were classified into academic audience sectors. We manually classified each of these 73 accounts as either academic or nonacademic by examining self-disclosed information in each user’s bio. If a bio was empty or ambiguous, we performed a Google search for the user’s screen name and/or username to determine whether they had an online presence associated with an academic institution. We determined 68 accounts belonged to individual academics (graduate students, postdocs, staff scientists, or professors), academic labs, university departments, or university-affiliated academic organizations. Further, 3 accounts (2 automated bots and 1 human-curated account) were dedicated to posting tweets about the latest papers in the field of biophysics/microscopy and were primarily followed by academics. One other account belonged to a company that manufactures and sells microscopes and ostensibly has a customer base (and thus, Twitter following) consisting of primarily scientists. This left only a single account that we could not unambiguously classify as an academic account, though deeper examination found that many of this account’s followers work within the field of biophysics and microscopy, indicating they also possess an affiliation with this academic network. We conclude that Altmetric misclassified over 50% of the academic (or academic-affiliated) accounts as “members of the public,” whereas our method, at worst, only misclassified a single user. This validation example demonstrates that our method of inferring audience demographics is potentially much more accurate at classifying academic-affiliated accounts than the Altmetric method.

### Inferring the characteristics of nonacademic audience sectors

A commonly cited advantage of altmetrics over traditional scientometrics is that altmetrics are capable of capturing information about engagement from lay audiences, which could be useful for evaluating dimensions of research productivity and scholarship that are often overlooked. For example, such information may guide researchers in describing and quantifying the broader impacts of their work in grant applications, enable new citizen science collaborations (such as fold-it, a crowdsourced computer game where players predict protein structures [42]), or crowdfunded research (such as the approximately 1,000 research projects that have been successfully funded on the scientific crowdfunding platform, experiment.com), and provide concrete evidence of researchers’ individual science communication and public engagement efforts.

To this end, we examined the nonacademic audience sectors inferred by our topic modeling analysis for the 4 preprints used as case studies in **Fig 1**. As with the inferred academic audience sectors, the characteristics of nonacademic audience sectors often aligned intuitively with the topic of the preprint. “DeepPoseKit, a software toolkit for fast and robust animal pose estimation using deep learning,” by Graving and colleagues [36], included nonacademic audience sectors associated with video game developers (topic 2), business applications of artificial intelligence (topics 6 and 9), and graphic designers (topic 11) (**Fig 1A**), presumably because this research has implications for more realistic computational rendering of physiological properties and organic movement. “Engineering Brain Parasites for Intracellular Delivery of Therapeutic Proteins,” by Bracha and colleagues [37], included nonacademic audience sectors we ascribe to be primarily associated with biotechnology companies (topics 2, 8, 9, and 10), aligning with the paper’s focus on bioengineering, as well as an audience sector associated with news media (topic 3), suggesting this preprint caught the attention of science journalists (though we note that at the time we analyzed these data, Altmetric has not indexed any news media coverage that cites this preprint) (**Fig 1B**). Other preprints showed a greater variety of nonacademic audience sectors. “Sex differences in gene expression in the human fetal brain,” by O’Brien and colleagues [38] had a particularly diverse array of nonacademic audience sectors, including groups associated with blockchain technology and cryptocurrency (topic 2), veganism and animal rights (topic 3), video games (topic 4), right-wing politics (topics 5, 6, and 7), and science fiction and fantasy writers (topic 10). Other audience sectors captured groups of individuals that shared a common language, specifically Arabic (topic 8) and Spanish (topics 11 and 12), indicating this preprint had a culturally and geographically diverse audience (**Fig 1C**). “How face perception unfolds over time,” by Dobs and colleagues [39] included nonacademic audience sectors associated with right-wing politics (topics 1, 2, and 12), Spanish-speaking communities (topics 3, 6, and 9), business and marketing (topics 4 and 10), mental health professionals and advocates (topic 7), sports (topic 8), and veganism and animal rights (topic 11) (**Fig 1D**).

### Measuring engagement from political partisans

As exemplified by our analysis of O’Brien and colleagues [38] and Dobs and colleagues [39] (**Fig 1C and 1D**), many nonacademic audience sectors included keywords signaling political ideology/affiliation such as “republican,” “conservative,” “democrat,” or “liberal”; hashtags such as “#MAGA” and “#KAG”, (acronyms for “make America great again” and “keep America great,” used by Donald Trump and his populist supporters throughout his campaign and presidency) and “#resist” (used primarily by members of the Democratic party in the US who critically oppose Donald Trump); and coded emoji such as 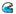 (a common symbol used by US liberals to show support for a “blue wave” of Democratic candidates in the 2018 midterm elections) or 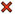 (used by conservatives to signal their claim that right-wing users have been unfairly subjected to censorship on social media [43]). The presence of these words, hashtags, and emoji demonstrate that many nonacademic audience sectors consist of users who primarily self-identify through their political ideologies rather than professional affiliation or personal/recreational interests and thus might be especially interested in the political implications of the research at hand.

**Fig 3:**
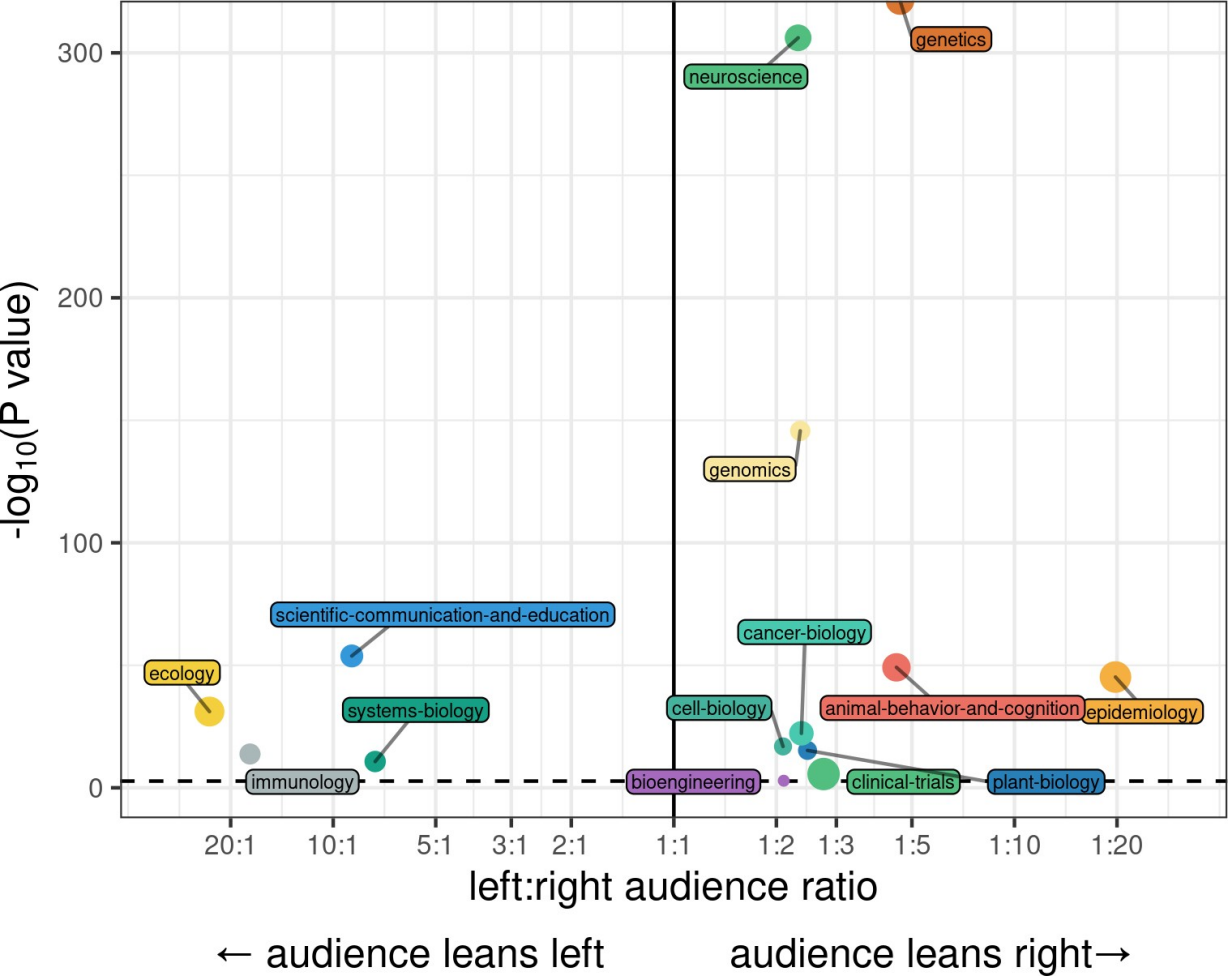
Political skew of nonacademic audience sectors by bioRxiv category. The x-axis shows the ratio between the estimated sizes of left-wing audience sectors (associated with the hashtag “#resist”) and right-wing audience sectors (associated with the hashtag “#maga”) among all tweets referencing preprints in a given bioRxiv category. Data shown are for the 14 bioRxiv categories where this ratio exceeds 2:1 or 1:2. The y-axis shows the -log10 *p*-value of a chi-square test for whether the sizes of these audience sectors match an underlying null distribution, assuming 62.5% of users lean left and 37.5% of users lean right, based on a recent poll of US Twitter users’ political ideologies. Preprint categories with statistically significant differences (after Bonferroni multiple testing correction) are annotated above the dashed line. The size of each point indicates the total number of users affiliated with political audience sectors for that category. Data for the information depicted in this figure are available at https://github.com/carjed/audiences.

To coarsely assess how preprints’ audience sectors polarize on the political spectrum (specifically within the United States, given that the US contains the largest base of Twitter users), we compared the estimated size of the audience sectors whose top 30 associated keywords contain the hashtag “#resist” (an indicator of left-of-center political affiliation in the US) to the estimated size of the audience sectors whose top 30 associated keywords contain the hashtag “#maga” (an indicator of right-of-center political affiliation in the US) [44] (Materials and methods). The political polarization of these audience sectors tended to vary by research area—tweets citing preprints in 4 bioRxiv categories (ecology, immunology, scientific communication/education, and systems biology) were significantly enriched for users whose follower networks skewed to the political left (by a ratio greater than 2:1), with the greatest discordance among ecology preprints, where left-leaning audiences outnumbered right-leaning audiences more than 20 to 1 (**Fig 3**). In contrast, 10 categories (most notably, genetics, genomics, neuroscience, epidemiology, and animal behavior and cognition) exhibited audiences that skewed to the political right (**Fig 3**). We observed similar patterns of polarization when audiences were dichotomized by the aforementioned politically coded emoji (**S4 Fig**). Other bioRxiv categories (e.g., synthetic biology, bioinformatics, biophysics, zoology) either showed no significant skew in their audiences’ political orientation after multiple testing correction, or could not be evaluated because no preprints in that area were found to have audience sectors corresponding to one political audience or another.

### Quantifying engagement with biological research by white nationalists

Further analysis of politically aligned audience sectors revealed that many of these were associated with extreme right-wing political ideologies, with 85 preprints exhibiting at least one audience sector that included the keyword “nationalist” in the top 30 keywords. In contrast, we found no indications of overtly far-left ideologies (e.g., communism) pervading the audience sectors we characterized as “left-wing.” A minority (13) of these preprints appeared to be capturing audience sectors affiliated with nationalism in the country of India, but most instances of “nationalist” audience sectors appeared to pertain specifically to white nationalist communities in the US, with 70 of the remaining 72 preprints exhibiting audience topics containing both the keywords “white” and “nationalist,” and 69 of these also containing the keyword “American” (e.g., topic 5 in O’Brien and colleagues, **Fig 1C**). Although these white nationalist audience sectors account for only 0.24% of the total audience across all 1,800 preprints, these sectors were detected in only 6 bioRxiv categories: animal behavior and cognition, bioinformatics, evolutionary biology, genetics, genomics, and neuroscience. This enrichment for white nationalist audience sectors was strongest in the categories of animal behavior and cognition and genetics, in which these sectors accounted for 1.53% and 1.51% of the total audiences of these categories, respectively—more than 6 times higher compared with the overall average across all preprints we analyzed. Moreover, among the 70 preprints that exhibited white nationalist audience sectors, these sectors comprised a median of 7.2% of the total audience, and a maximum of 25% of the total audience.

To better quantify the extent of this engagement and confirm that this result was not an artifact of the LDA model we used, we applied an orthogonal analysis of political affiliation by examining patterns of network homophily (i.e., the number of mutual followers shared [22]) between each user that tweeted about a preprint and a curated reference panel of 20 prominent white nationalist accounts on Twitter. Across the 331,696 users analyzed, the median network homophily of individual users was 0.1%, with 95% of users exhibiting homophily levels of <1%. Thus, we considered any user with far-right network homophily greater than 2% (corresponding to at least a 2-fold larger degree of far-right network homophily than 95% of users in the dataset) to be affiliated with this network. We emphasize that this is a strictly quantitative affiliation, and we make no claims about the interpersonal or ideological association between an individual user and white nationalist communities on Twitter, simply that they share substantially more followers in common with prominent white nationalists on Twitter than the majority of users analyzed.

**Fig 4:**
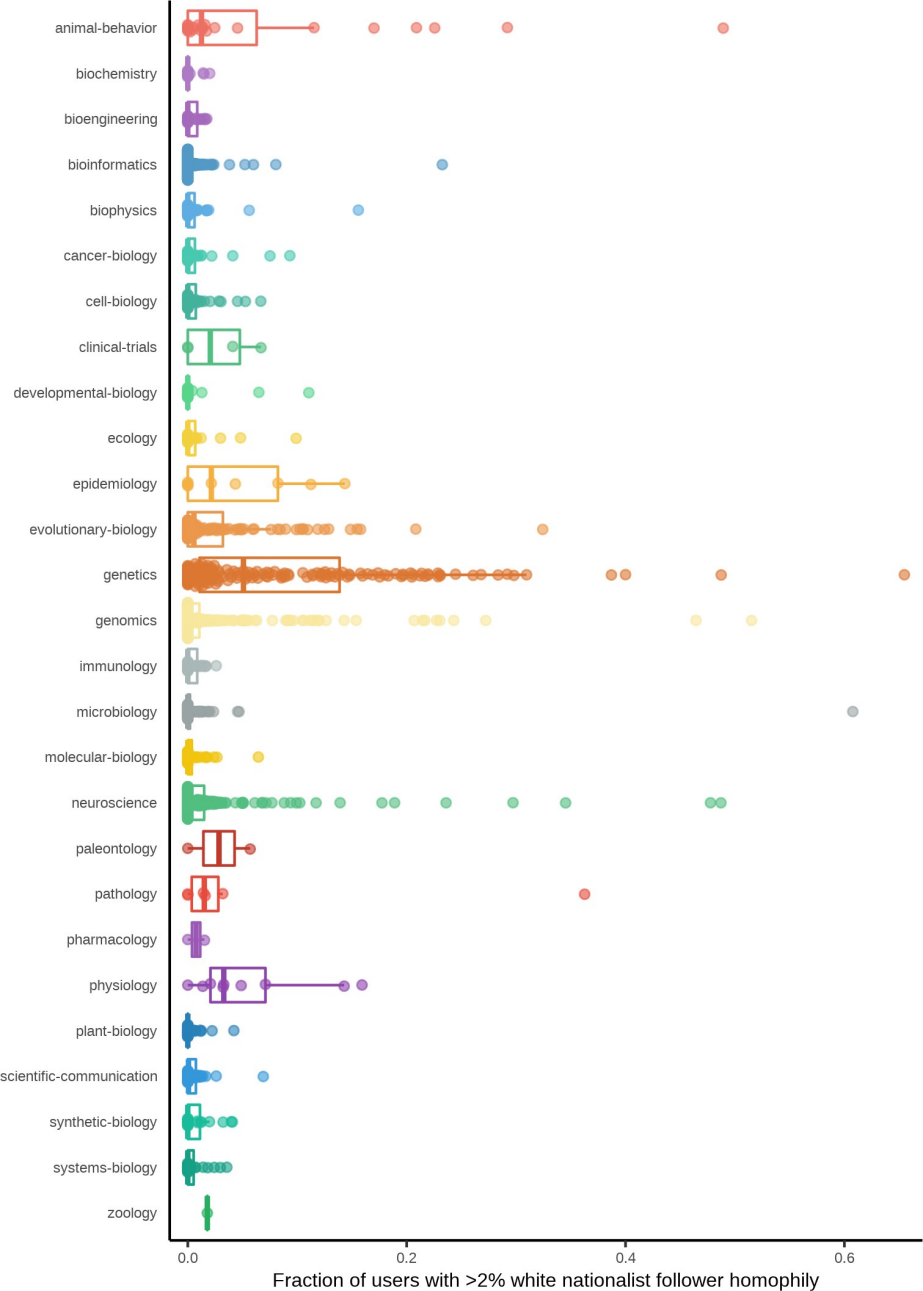
Distributions of white nationalist homophily by category. Each point represents a single preprint, and the position on the y-axis indicates the proportion of users who tweeted about that preprint whose follower network homophily with the white nationalist reference panel is greater than h = 2%. Boxplots summarizing the distributions of these proportions per bioRxiv category are shown beneath each set of points. Data for the information depicted in this figure are available at https://github.com/carjed/audiences, and an interactive version of this figure can be accessed at https://carjed.github.io/audiences.

We next specified varying thresholds of white nationalist network homophily (2%, 5%, 10%, and 20%) and for each preprint, counted the number of users whose median homophily with the white nationalist reference panel exceeded each threshold (**Fig 4; S5 Fig**). As a baseline, we found that a majority (1,286; 71.4%) of the preprints analyzed had audiences in which the fraction of users with >2% follower network homophily with the white nationalist reference panel was negligible (<1% of tweets referencing the preprint), indicating that engagement from white nationalist-affiliated users cannot be simply explained as a normative aspect of any research discussed on Twitter.

Many of the remaining 514 preprints had an even stronger exposure to white nationalist-affiliated audience sectors than indicated by the audience topics alone: 182 of these (10.1% of all preprints analyzed) had >5% of users each with >2% white nationalist network homophily with the reference panel, 14 of which had >30% of users with >2% white nationalist network homophily. Preprints with the strongest enrichment of such users generally occurred in bioRxiv categories, in which we detected explicit white nationalist–associated audience sectors (animal behavior and cognition, evolutionary biology, genetics, genomics, and neuroscience), though we note that we also detected at least one preprint in which >5% of tweets came from users with >2% white nationalist network homophily in 19 of the 27 bioRxiv categories (**Fig 4**).

The 182 preprints that showed a high level of engagement from white nationalist-affiliated accounts (>5% of users, each with >2% far-right network homophily) differed significantly from the remaining 1,618 preprints on several Altmetric indicators (number of tweets received, number of mentions in mainstream news sources, Altmetric Attention Score, estimated academic audience fraction) but did not differ significantly in the number of citations received in the scientific literature (**Table 1**). The preprints attracting extremist audiences also tended to be slightly older than other preprints—this difference was statistically significant, but the group medians only differed by approximately 6 weeks, so it is unlikely that the increased number of tweets and mainstream news references can be explained by these preprints having had substantially more time to accumulate such attention (**Table 1**).

**Table 1:** Comparison of altmetric and bibliometric indicators for preprints attracting extremist and nonextremist audiences.

Metric	Median value for preprints attracting extremist audiences (*N* = 182)	Median value for all other preprints (*N* = 1,618)	*p*-value of Wilcoxon rank-sum test
Number of tweets received	136	110	3.75e-05
Mentions in mainstream news sources	4	1	5.69e-04
Altmetric Attention Score	98.4	69.2	2.83e-09
Academic Audience Fraction (our estimate)	0.76	0.94	1.04e-48
Academic Audience Fraction (Altmetric’s estimate)	0.35	0.51	3.69e-42
Number of literature citations	3	3	0.85
Date posted to bioRxiv	September 18, 2018	November 4, 2018	4.52e-03

## Discussion

Our analyses demonstrate that the audiences engaging with scientific research on Twitter can be accurately classified into granular, informative categories across both academic and nonacademic communities purely by examining how each user’s followers self-identify in their Twitter biographies. This audience partitioning approach enables more accurate appraisal and contextualization of an article’s exposure to various communities on Twitter, reveals patterns of interaction from political partisans that may reflect immediate policy implications of contemporary research, and quantifies troubling trends of engagement with the scientific literature from users affiliated with far-right white nationalist networks.

It is important to acknowledge that the results for a given preprint represent a single temporal snapshot of its Twitter audience—additional users may discover and tweet about the preprint, follower networks are constantly shifting, and users may change their biographies, delete their tweets, deactivate their accounts, or be suspended at any time. The constant evolution of social media data may mean that the conclusions from our present study are only valid at a particular point in time, so tracking these trends longitudinally might be necessary to separate signals of ephemeral attention from evidence of long-term societal impacts. There are numerous other untapped features of social media data that could be useful for parsing characteristics of a preprint’s audience, such as a sentiment analysis of the tweets referencing the preprint, indexing of users who like or reply to tweets referencing the preprint, or the patterns of how original tweets referencing the preprint accumulate retweets over time. Our method might also be applied to characterize Twitter audiences by groups of networked users other than a user’s followers, such as who each user follows or their reciprocal follower relationships. However, for the purposes of this study, we consider “follower” sets to be a more reliable indicator of a preprint’s audience, as it is ultimately a user’s followers (not who they follow) who are exposed to the preprint when the user tweets about it.

Our study focused exclusively on bioRxiv preprints because of the accessibility of article-level metadata and breadth of research topics covered, but this audience segmentation strategy could be extended to evaluate the social media audiences of research appearing in peer-reviewed journals, too. For preprints that are eventually published in peer-reviewed journals, we could even compare and contrast the audience sectors of the manuscript in both its preprint and peer-reviewed forms to investigate how the peer review and editorial processes influence audience composition. A priori, we may expect the pre- and post-publication audiences to be quite similar, but many factors (press releases, journal prestige, major revisions made to the manuscript after peer review, open-access status of the paper, stochastic effects of being tweeted by an influential Twitter users, etc.) could influence the number and balance of stakeholders interested in a paper. As a proof of principle, we performed our audience segmentation on 8 manuscripts included in our preprint dataset that were eventually peer-reviewed and published in the Springer Nature family of journals in the area of genetics/genomics. In their peer-reviewed form, these manuscripts tended to receive more tweets, have smaller academic audience fractions, and received relatively less attention from users with white nationalist network homophily (**S6 Fig**). This suggests that both scientists and users connected with far-right extremist communities are particularly engaged with preprints on Twitter, whereas other lay audiences are relatively underexposed to the preprint ecosystem. However, because of the extremely biased nature of this small sample, a more systematic investigation of these trends is warranted. More generally, such data could help journals identify strengths and growth opportunities for their editorial/publishing practices, assist authors in making informed decisions about where to submit and whether to preprint their work, and guide readers toward research that is most relevant to their interests.

Another source of data that is largely ignored in the altmetrics literature is “secondary engagement” events—tweets that do not link directly to a research article but instead cite news articles, blog posts, and other online sources that reference or discuss the research. These secondary engagement events are not indexed by altmetric data brokers, but given that most lay audiences learn about new scientific research through news media [1], it is likely that such posts are heavily enriched for nonacademic audiences and thus more informative of potential societal impacts. For example, the paper “Loci associated with skin pigmentation identified in African populations” [45] received 374 tweets from 323 users as of November 5, 2019, according to Altmetric. This study was covered by science journalists Ed Yong in The Atlantic [46] and Carl Zimmer in The New York Times [47] (in addition to over 40 other news outlets, according to Altmetric). Tweets linking to news articles posted by Yong, Zimmer, and their respective publishers alone received over 700 retweets, far outnumbering the tweets that link directly to the research article.

### Scientists are the primary drivers of social media engagement

We estimate that academic-affiliated accounts comprise the majority of the Twitter audience for over 95% of the bioRxiv preprints analyzed, suggesting that most social media discussion of preprints remains confined to the academic community. This stands in sharp contrast to Altmetric’s audience segmentation estimates for the same set of preprints, which imply that less than half of the preprints analyzed have majority academic-affiliated audiences on Twitter. Our results suggest that Altmetric tends to severely underestimate the size of these academic audiences. If we assume our method is a perfect classifier of these audiences, this indicates Altmetric’s median misclassification rate for these audiences is approximately 40%. We do not claim our method is a perfect classifier of academic audience sectors, but these discrepancies certainly highlight the need for greater transparency in the methodologies used by commercial altmetric data brokers and assurance that their altmetric indicators are situated in an informative, accurate context.

These results, coupled with the fact that scientists on Twitter are particularly susceptible to the effects of network homophily (a recent paper estimated that faculty members in ecology and evolutionary biology typically have a Twitter following comprised mostly [55%] of other scientists [9]), also leads us to conclude that most discussions of bioRxiv preprints on social media are often simply an online extension of the broader academic research ecosystem. This conclusion challenges a recent study that estimated less than 10% of tweets referencing scientific publications originated from accounts that conveyed “curated, informed perspectives” on the research, whereas most tweets in that study appeared to originate from automated bots or accounts the authors characterized as duplicative, mechanical, or “devoid of original thought” [5] (note that this study only considered papers in dentistry journals). To the contrary, our finding that scientists are the largest audience sector would suggest that the opposite is true, and the vast majority of tweets referencing bioRxiv preprints represent curated, informed perspectives from subject matter experts. Although we cannot guarantee that every scientist on social media is immune to mechanical tweeting or performative attempts to build social capital (colloquially known as “clout” on Twitter) with their peers, we are optimistic that most are doing so in an honest attempt to stay abreast of the latest work in their fields and broadcast their informed opinions (or the opinions of trusted colleagues, via retweets) to their followers.

Nevertheless, the overwhelming ubiquity of academics in the audiences of bioRxiv preprints demonstrates that most preprints ultimately appear to be receiving fairly limited engagement from lay audiences. This presents an opportunity for motivated scientists to self-audit their own network homophily, build a following beyond their professional bubble, and use the platform for spreading scientific information to the broader public. If funding agencies and policymakers continue to prioritize societal impact and altmetrics as a desirable outcome of scientific research and demand evidence of impactful scholarship, it may be beneficial to explicitly incentivize such public engagement and reward researchers who develop and maintain a public-facing presence on social media.

Our analysis of Graving and colleagues provides a motivating example of how our social media audience segmentation approach can help identify potential downstream societal impacts of research output and recognize the efforts of individual researchers (**Fig 1A**). This preprint introduced a software program called DeepPoseKit that uses deep learning to understand the dynamics of how animals sync and swarm together [36]. Of the 24% of Graving and colleague’s audience that we classified as nonacademic, certain sectors appeared to be associated with video game developers and graphic designers, perhaps indicating this research has immediate economic and cultural applications through the visual arts. Much of the total engagement (approximately 270 retweets) surrounding this preprint stemmed from a single tweet posted by the first author of the study, Jacob Graving, who provided a brief summary along with an animated GIF showing how the program tracks the movements of a swarm of locusts (https://twitter.com/jgraving/status/1122043261777076224). Neither the preprint itself nor Graving’s tweet alluded to any prospects of economic, cultural, environmental, or social applications, and both focused solely on the ethological aspects of their software (though we note that the authors disclose in their manuscript that they received unrestricted funding from graphics card manufacturer Nvidia, which may partially explain why this preprint was of interest to video game developers). Our audience segmentation of this preprint demonstrates that researchers are fully capable of capturing the attention of lay audiences simply by maintaining a presence on Twitter and creatively communicating their work.

### Uncovering patterns of political engagement

Many preprints exhibited audience sectors characterized by overtly political terminology, typically pertaining to the political ecosystem of the US. Intriguingly, the fields that attracted these politically oriented audience sectors rarely received equal bipartisan attention (as we might expect for research topics that have become battlegrounds of political disagreement in the US, such as climate change [48], or fields that typically transcend political boundaries, such as translational biomedicine [49]). Instead, when politically oriented audience sectors were present, they tended to polarize very strongly towards one end of the US political spectrum or the other.

Two categories of bioRxiv preprints stood out as attracting disproportionately left-leaning lay audiences: ecology and scientific communication and education. Many of the ecology preprints we analyzed dealt with aspects of climate change, a topic that receives far more positive attention and support from left-leaning political constituencies [49]. Similarly, the scientific communication and education category includes several preprints that address issues of equity, diversity, and inclusion in academic environments, which are also a prominent feature of left-wing politics. Preprints in genetics, neuroscience, and animal behavior and cognition attracted a disproportionately stronger presence from right-leaning lay audiences. These preprints often involved research pertaining to human population history and the genetic and neurological architecture and evolution of sociobehavioral traits, suggesting such research is seen by these audience sectors as especially relevant to right-wing political ideologies. A cursory examination of tweets referencing these preprints indicates these right-wing lay audiences generally view this research through a positive lens. For example, a conservative political scientist with over 80,000 followers tweeted a reference to “Genetic Associations with Mathematics Tracking and Persistence in Secondary School” by Harden and colleagues [50] and interpreted the conclusions of the paper as follows:

*Want an example of how PGS* [polygenic scores] *can inform policy issues*? *Voila*. (https://twitter.com/charlesmurray/status/1114536610266267649).

We strongly emphasize that the authors of this particular preprint (or any other, for that matter) do not necessarily endorse the interpretations of audiences that find it interesting and relevant, but this is a concrete example of how easily research can be appropriated in arguments for or against specific policies.

Upon further investigation of the audience sectors we defined as “right-wing,” we found many such sectors were also characterized by keywords indicative of extreme far-right themes and ideologies, such as white nationalism or a popular anti-Semitic conspiracy theory known as “QAnon” that originated on the 4chan and 8chan message boards [51]. The presence of these “extremist” audience sectors led us to confirm that many users engaging with preprints exhibit unusually high levels of network homophily with prominent white nationalists on Twitter. Recent studies and commentaries from journalists, scientists, and scholarly societies have expressed concerns about the recent resurgence of white nationalism in the US and Europe and their use of scientific research to promote discredited racist ideologies [30–32,52–55]. Our study rigorously quantifies the extent of these qualitative observations—by one heuristic, over 10% of the preprints we analyzed received at least 5% (and in extreme cases, over 50%) of their total tweets from accounts that showed a high degree of network homophily with prominent white nationalists. We must strongly emphasize that we do not claim any particular user found to be associated with audience topics pertaining to white nationalism and/or exhibiting higher than usual levels of network homophily with white nationalists is ideologically aligned with such movements, merely that a nontrivial fraction of their Twitter followers likely are.

Naturally, these results elicit questions about how scientists should respond. According to a recent news report, many scientists engaging in basic research that intersects socially and politically sensitive topics have expressed that they are reluctant to confront politicized misappropriation/misinterpretation of their work or avoid doing so because they feel incapable of successfully communicating the complexities of their research to nonexpert audiences [31]. However, recent research has demonstrated that the reach of science denialism is actually amplified when subject matter experts and advocates do not intervene, but the spread of science denialism was significantly attenuated when experts and advocates responded with factual information or addressed the faulty rhetorical techniques of denialists [56].

Even so, the tactics proven to be effective at stemming the spread of science *denialism* may not translate well to the task of stopping the spread of science *misappropriation*. As noted by Panofsky and Donovan [53], white nationalists—unlike right-wing deniers of the reality of climate change and vaccine efficacy [48,57,58]—are not necessarily filtering scientific information through a denialist mindset or extreme misinterpretations but rather by processing through racist cognition. Panofsky and Donovan go on to conclude that “challenging racists’ public understanding of science is not simply a matter of more education or nuance, but may require scientists to rethink their research paradigms and reflexively interrogate their own knowledge production” [53]. We anticipate the results of our study will motivate researchers to engage with these uncomfortable yet unavoidable challenges of scientific inquiry and communication.

## Materials and methods

### Data collection

Using the Rxivist API [33], we collected metadata for 1,800 bioRxiv preprints, considering any preprints ranked within the top 1,000 by total number of downloads or total number of tweets that received 50 or more tweets from unique Twitter users. Note that this is not an exhaustive collection of the most highly tweeted preprints, as preprints posted prior to February 2017 were excluded from the rankings by tweet count (we expect that many of these older, highly tweeted preprints were captured in the ranking of the top 1,000 preprints by download count, but there are likely some older preprints that received >50 tweets but few downloads).

For each preprint, we queried the Crossref Event Data API for all documented tweets and retweets that referenced that paper/preprint. In instances in which tweets referencing a paper were not indexed in the Crossref event database, we used the *rvest* R package to scrape equivalent information from the preprint’s Altmetric page. Specifically, we collected (1) the handle of the user, (2) the timestamp at which they (re)tweeted the article, (3) the text of the (re)tweet, and (4) whether the event was a retweet or the user who originally posted the tweet. Tweets from private users or users with 5 or fewer followers were excluded, to avoid making inference on empty or overly sparse data. We used the *tweetscores* R package [22] to query the Twitter API for the follower metadata (account handles and biographies of up to 10,000 followers per user) of each of the *N* unique users that (re)tweeted a given preprint. Because of the Twitter developer agreement, we are unable to provide the raw data used in these analyses; the R code we developed to scrape the data is available at http://github.com/carjed/audiences.

### Topic modeling

For each of the *N* users that (re)tweeted a reference to a given preprint, we concatenated the biographies and screen names of their followers into a single “document,” representing a list of all the words contained in the Twitter biographies/screen names of that user’s followers. We cleaned each document to remove punctuation and common stopwords (e.g., “a”, “the”, “is”) from 13 languages using the *tm* R package [59]. Note that we did not preprocess the corpus with a stemming/lemmatization algorithm, as doing so does not meaningfully improve model fit and coherence and can destabilize the inferred topics [60,61]. We translated emoji that occurred in the follower documents into a unique single word string according to the official emoji shortcode, taken from https://emojipedia.org, and prepended with the string “emoji” to create an alphanumeric word, e.g., the microscope emoji (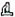) was translated to “emojimicroscope” to ensure it was not excluded during preprocessing of the corpus. Similarly, we translated hashtags to replace the “#” symbol with “hashtag”, e.g.,“#microscope” was translated to “hashtagmicroscope.” For the *W* unique words observed across all the *N* follower documents, we then generated an *NxW* document term matrix, enumerating the frequencies of each word in each follower document.

We used the *lda* R package to apply an LDA model to this document term matrix, representing each of the *N* documents as a mixture of *K* discrete topics (where *K<<N*). For consistency, we specified K = 12 for all preprints. The LDA model estimates 2 sets of relevant hyperparameters: *θ*_*i* = 1,…,*N,k* = 1,…*K*_, indicating the probability of topic *k* occurring in the follower document for user *i*, and *ϕ*_*w* = 1,…,*W,k* = 1,…*K*_, indicating the probability of word *w* occurring in topic *k*. Each topic is thus characterized by words that frequently co-occur, and each follower document is summarized as a set of dosages corresponding to the probabilistic topic assignments.

### Estimation of academic audience fractions

For a given preprint, we estimated the size of the audience that were academics by identifying topics containing keywords we determined to correspond to academic careers/environments (e.g., “university”, “phd”, “postdoc”, “professor”, “fellow”), then summing the theta parameters of these topics over the N users. Formally, we define the estimated academic audience fraction as:

$$f_{academic}=\frac{1}{N}\sum_{i=1}^{N}\sum_{k=1}^{K}\theta_{i,k}I(topic_{k}∋\{"university","phd","postdoc","professor",\ldots \})$$

The estimated nonacademic audience fraction is thus:

$$f_{non-academic}=1-f_{academic}$$

### Political polarization analysis

Similar to our estimation of academic/nonacademic audience sizes, we estimated the sizes of left-leaning and right-leaning audiences for each preprint by identifying audience sectors containing politically coded terms (either the hashtags “#resist” and “#MAGA”) then summing these topic probabilities across the N users that tweeted about the preprint:

$$N_{left-leaning}=\sum_{i=1}^{N}\sum_{k=1}^{K}\theta_{i,k}I(topic_{k}∋\{"\#resist"\})$$

$$N_{right-leaning}=\sum_{i=1}^{N}\sum_{k=1}^{K}\theta_{i,k}I(topic_{k}∋\{"\#MAGA"\})$$

For the majority of preprints, one or both of these estimates were 0, which precluded our ability to test for political polarization among the audiences of individual preprints. Instead, we summed these estimates across preprints of a given bioRxiv category (e.g., all preprints in our dataset in the category “science communication and education”) to evaluate whether preprints of a given field attracted predominantly left-leaning or right-leaning audiences on Twitter. According to a recent Pew survey [62], 60% of US adult Twitter users identify as “Democrat or lean Democrat,” and 35% of US adult Twitter users identify as “Republican or lean Republican,” so we based our tests on a null expectation that 62.5% of the politically affiliated audience leans left and 37.5% of the politically affiliated audience leans right. For each bioRxiv category, we then tested whether the sizes of left-leaning and right-leaning audience sectors differed from these expected frequencies using a chi-square test.

### Network homophily analysis

We curated a reference set of 20 organizations and individuals associated with far-right white nationalist ideologies. For each of these accounts, we then scraped their list of followers. Then for each preprint analyzed, we calculated the fraction of each referencing user’s followers that were also following each of these 20 accounts, taking the median of these 20 scores as an estimate of that user’s network homophily with the reference panel. We summarized these individual-level homophily scores on a per-preprint basis by calculating the fraction of individuals with homophily score >*h*, varying *h* at 4 different stringency thresholds {2%, 5%, 10%, and 20%}.

## Supporting information

S1 Fig Distributions of preprint audience fractions by topic coherence. For each preprint, we identified any audience sectors whose list of top 30 associated keywords included a keyword matching the focal category of that preprint (e.g., an audience sector containing the keyword “plant” for a preprint submitted in the “plant biology” category, or an audience sector containing the keyword “genetic” for a preprint submitted in the “genetics” category), then calculated the fraction of that preprint’s audience corresponding to those matching audience sectors. The distributions of these audience fractions are plotted as separate histograms for each bioRxiv category (note that we excluded the bioengineering and zoology categories, as we did not detect any matching audience sectors among the preprints in these categories). Data for the information depicted in this figure are available at https://github.com/carjed/audiences.
(TIFF)

S2 Fig Comparison of academic audience fractions estimated by Altmetric versus our estimates, separated by bioRxiv category. Each point represents an individual preprint, with Altmetric’s estimated academic audience fraction shown on the x-axis, and the academic audience fractions estimated by our topic modeling approach shown on the y-axis. The loess-smoothed curve fit to the data in each panel indicates a nonlinear relationship between these 2 sets of estimates. The size of each point indicates the total number of tweets referencing that preprint. Data for the information depicted in this figure are available at https://github.com/carjed/audiences, and an interactive version of this figure can be accessed at https://carjed.github.io/audiences.
(TIFF)

S3 Fig Relationship between estimated academic audience fraction and number of tweets received by each preprint. Each point indicates one of the 1,800 preprints in our dataset, with the estimated academic audience fraction along the x-axis and the number of (re)tweets on a log-scale on the y-axis. Data for the information depicted in this figure are available at https://github.com/carjed/audiences.
(TIFF)

S4 FigPolitical skew of nonacademic audience sectors by bioRxiv category.The x-axis shows the fold difference between the estimated sizes of right-wing audience sectors (associated with the 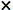 emoji) and left-wing audience sectors (associated with the 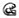 emoji) among all tweets referencing preprints in a given bioRxiv category. The y-axis shows the -log10 *p*-value of a chi-square test for whether the sizes of these audience sectors match an underlying null distribution, assuming 62.5% of users lean left and 37.5% of users lean right, based on a recent poll of US Twitter users’ political ideologies. Preprint categories with statistically significant differences (after Bonferroni multiple testing correction) are annotated above the dashed line. The size of each point indicates the total number of users affiliated with political audience sectors for that category. bioRxiv categories with nonsignificant differences are excluded from this plot. Data for the information depicted in this figure are available at https://github.com/carjed/audiences.(TIFF)

S5 Fig Distributions of white nationalist homophily by category. Each point represents a single preprint, and the position on the y-axis indicates the fraction of users who tweeted about that preprint whose follower network homophily with the white nationalist reference panel is greater than (**a)** h = 2%, (**b)** h = 5%, (**c)** h = 10%, and (**d)** h = 20%. Boxplots summarizing the distributions of these fractions per bioRxiv category are shown beneath each set of points. Data for the information depicted in this figure are available at https://github.com/carjed/audiences, and an interactive version of this figure can be accessed at https://carjed.github.io/audiences.
(TIFF)

S6 Fig Comparison of audience altmetrics for select bioRxiv preprints and their peer-reviewed format after publication in Springer Nature journals. Each panel indicates a given metric, and each pair of bars indicates the values of that metric for a paper based on the Twitter audiences of its preprint form (red) and its peer-reviewed form (blue). Data for the information depicted in this figure are available at https://github.com/carjed/audiences.
(TIFF)

## Abbreviations

API: Application programming interface
LDA: latent Dirichlet allocation
