# Supplementary figures and images for: Quantifying and contextualizing the impact of bioRxiv preprints through automated social media audience segmentation
Source: PLoS Biol. 2020 Sep 22;18(9):e3000860. doi: 10.1371/journal.pbio.3000860 (PMC7508356; doi:10.1371/journal.pbio.3000860)

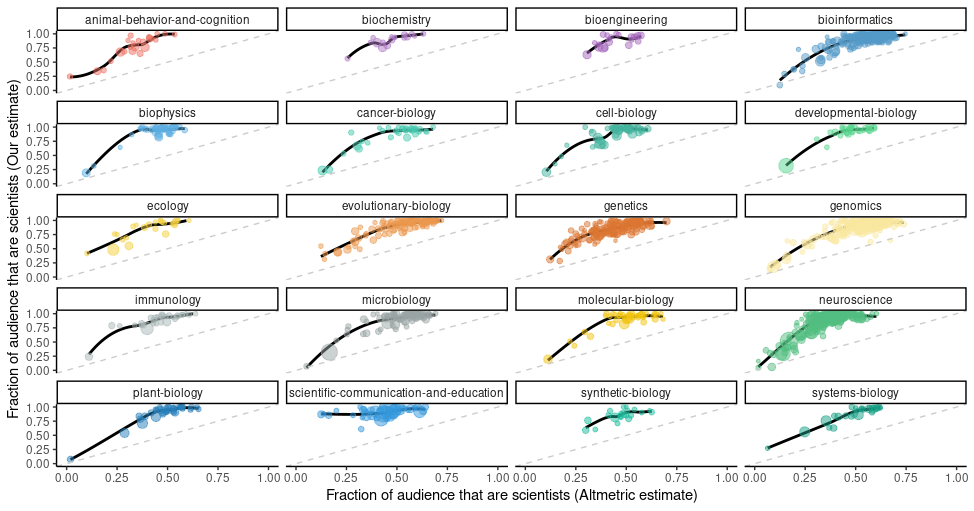

Supplement: S2 Fig — Each point represents an individual preprint, with Altmetric’s estimated academic audience fraction shown on the x-axis, and the academic audience fractions estimated by our topic modeling approach shown on the y-axis. The loess-smoothed curve fit to the data in each panel indicates a nonlinear relationship between these 2 sets of estimates. The size of each point indicates the total number of tweets referencing that preprint. Data for the information depicted in this figure are available at https://github.com/carjed/audiences, and an interactive version of this figure can be accessed at https://carjed.github.io/audiences. (TIFF) [file pbio.3000860.s002.tiff]

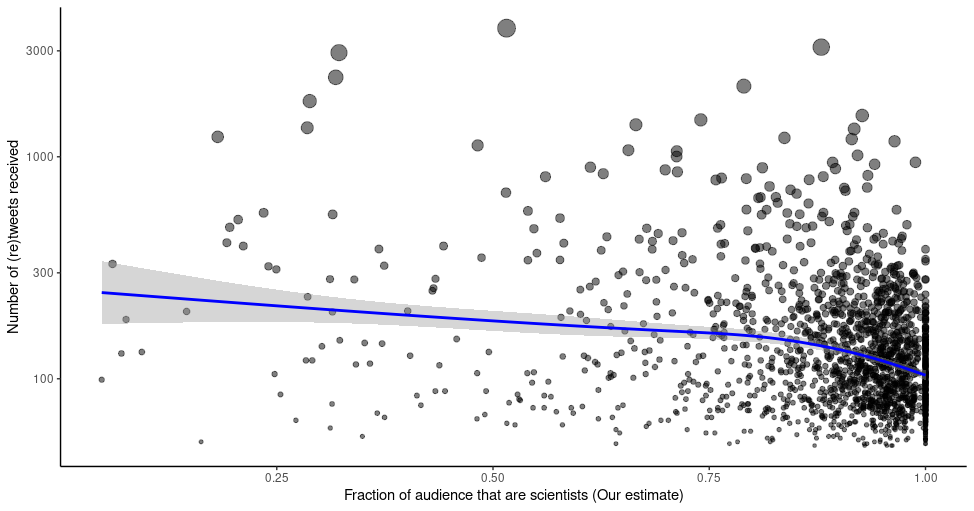

Supplement: S3 Fig — Each point indicates one of the 1,800 preprints in our dataset, with the estimated academic audience fraction along the x-axis and the number of (re)tweets on a log-scale on the y-axis. Data for the information depicted in this figure are available at https://github.com/carjed/audiences. (TIFF) [file pbio.3000860.s003.tiff]

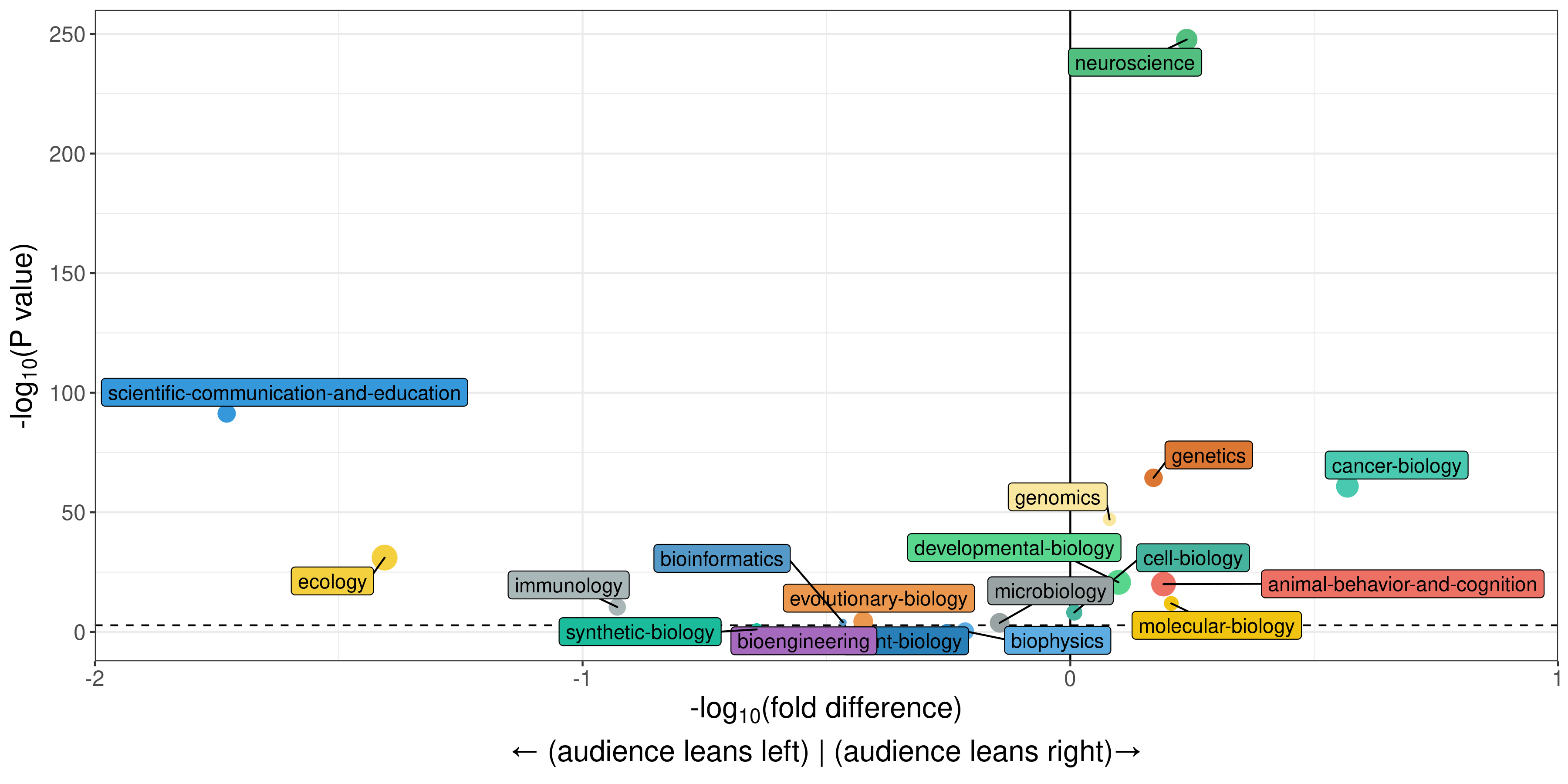

Supplement: S4 Fig — The x-axis shows the fold difference between the estimated sizes of right-wing audience sectors (associated with the emoji) and left-wing audience sectors (associated with the emoji) among all tweets referencing preprints in a given bioRxiv category. The y-axis shows the -log10 p-value of a chi-square test for whether the sizes of these audience sectors match an underlying null distribution, assuming 62.5% of users lean left and 37.5% of users lean right, based on a recent poll of US Twitter users’ political ideologies. Preprint categories with statistically significant differences (after Bonferroni multiple testing correction) are annotated above the dashed line. The size of each point indicates the total number of users affiliated with political audience sectors for that category. bioRxiv categories with nonsignificant differences are excluded from this plot. Data for the information depicted in this figure are available at https://github.com/carjed/audiences. (TIFF) [file pbio.3000860.s004.tiff]

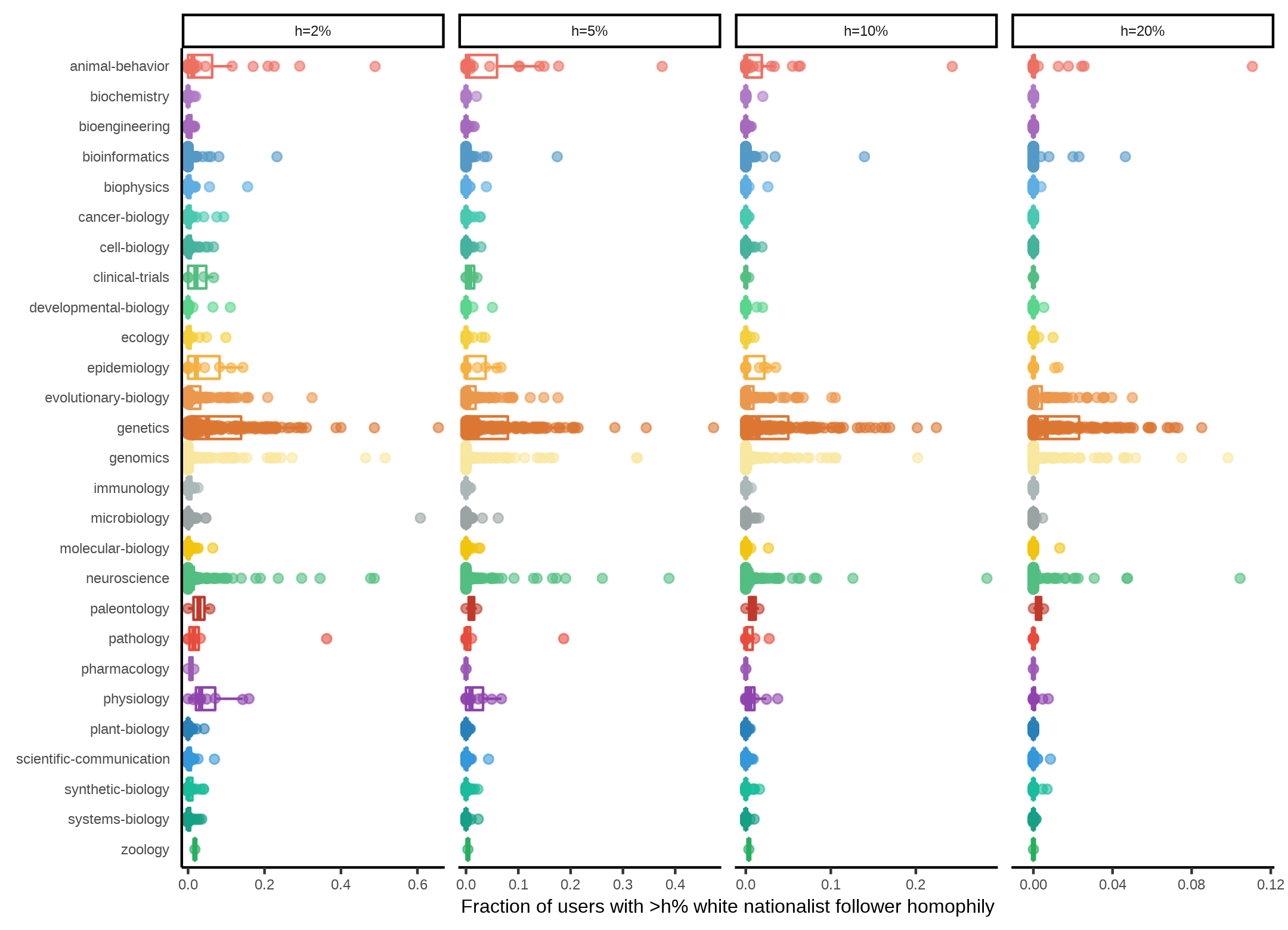

Supplement: S5 Fig — Each point represents a single preprint, and the position on the y-axis indicates the fraction of users who tweeted about that preprint whose follower network homophily with the white nationalist reference panel is greater than (a) h = 2%, (b) h = 5%, (c) h = 10%, and (d) h = 20%. Boxplots summarizing the distributions of these fractions per bioRxiv category are shown beneath each set of points. Data for the information depicted in this figure are available at https://github.com/carjed/audiences, and an interactive version of this figure can be accessed at https://carjed.github.io/audiences. (TIFF) [file pbio.3000860.s005.tiff]
